# Cutaneous metastases of lung cancer

**DOI:** 10.1002/ccr3.2331

**Published:** 2019-07-25

**Authors:** Yosuke Fukuda, Hatsuko Mikuni, Tetsuya Homma, Hironori Sagara

**Affiliations:** ^1^ Department of Medicine, Division of Respiratory Medicine and Allergology Showa University School of Medicine Tokyo Japan

**Keywords:** cutaneous metastasis, lung cancer, superior vena cava syndrome

## Abstract

Cutaneous metastases of lung cancer are rare. It was often painless and less likely to be noticed. Similar to SVC syndrome, cutaneous metastases may cause upper limb edema. We need to keep in mind that cutaneous metastases may account for the radiotherapy‐resistant upper limb edema.

A 72‐year‐old woman presented to the hospital with right arm edema. She was previously diagnosed as lung adenocarcinoma in the right upper lobe. Her right arm showed pitting edema and purple, painless multiple solitary nodules were found on the right side of her chest (Figure 1A). Physical examination and computed tomography findings (Figure 1B) revealed cutaneous metastases of lung cancer with superior vena cava (SVC) syndrome. Cutaneous biopsy was attempted, but the procedure was refused by the patient. She was treated with palliative radiation therapy against SVC syndrome, but the effect against edema was scarce.

**Figure 1:**
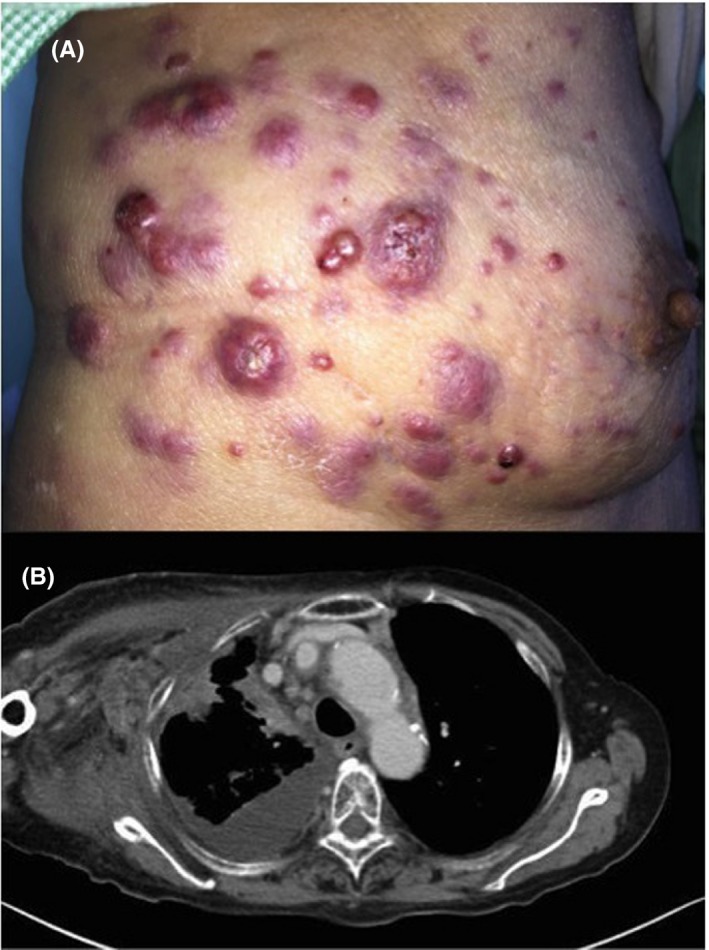
A, skin manifestations reveal multiple purple, solitary, and painless nodules suggesting malignancy. B, the image reveals stenosis of the superior vena cava

Reported cases of cutaneous metastases of lung cancer were 1.78% with poor prognosis.^1^ Cutaneous metastases accompanied with lymphangitis carcinomatosa shows various erythemas with upper limb edema,^2^ similar to SVC syndrome which is a major cause of upper limb edema occurring in patients with lung cancer. Systemic chemotherapy is a standard therapy for cutaneous metastases, but is rarely performed due to poor performance status with limited life expectancy. Other than SVC syndrome, clinicians need to keep in mind that cutaneous metastases may account for the radiotherapy‐resistant upper limb edema. Prompt diagnosis with treatment may enable better quality of life for the patient.

## CONFLICT OF INTEREST

Nothing to declare.

## AUTHORS' CONTRIBUTIONS

YF: performed clinical diagnosis, drafted and revised the manuscript. HM: performed clinical diagnosis. TH: reviewed the manuscript. HS: contributed to the final revision of the manuscript.
